# Ethereum Phishing Scam Detection Based on Data Augmentation Method and Hybrid Graph Neural Network Model

**DOI:** 10.3390/s24124022

**Published:** 2024-06-20

**Authors:** Zhen Chen, Sheng-Zheng Liu, Jia Huang, Yu-Han Xiu, Hao Zhang, Hai-Xia Long

**Affiliations:** 1 College of Information Science Technology, Hainan Normal University, Haikou 571158, China; cz221201@hainnu.edu.cn (Z.C.); lsz231209@hainnu.edu.cn (S.-Z.L.); hj221204@hainnu.edu.cn (J.H.); xyh221333@hainnu.edu.cn (Y.-H.X.); 2 Key Laboratory of Data Science and Smart Education, Ministry of Education, Hainan Normal University, Haikou 571158, China; 110058@hainnu.edu.cn; 3 College of Tourism, Hainan Normal University, Haikou 571158, China

**Keywords:** blockchain, Ethereum, phishing scam detection, data augmentation, DA-HGNN

## Abstract

The rapid advancement of blockchain technology has fueled the prosperity of the cryptocurrency market. Unfortunately, it has also facilitated certain criminal activities, particularly the increasing issue of phishing scams on blockchain platforms such as Ethereum. Consequently, developing an efficient phishing detection system is critical for ensuring the security and reliability of cryptocurrency transactions. However, existing methods have shortcomings in dealing with sample imbalance and effective feature extraction. To address these issues, this study proposes an Ethereum phishing scam detection method based on DA-HGNN (Data Augmentation Method and Hybrid Graph Neural Network Model), validated by real Ethereum datasets to prove its effectiveness. Initially, basic node features consisting of 11 attributes were designed. This study applied a sliding window sampling method based on node transactions for data augmentation. Since phishing nodes often initiate numerous transactions, the augmented samples tended to balance. Subsequently, the Temporal Features Extraction Module employed Conv1D (One-Dimensional Convolutional neural network) and GRU-MHA (GRU-Multi-Head Attention) models to uncover intrinsic relationships between features from the time sequences and to mine adequate local features, culminating in the extraction of temporal features. The GAE (Graph Autoencoder) concept was then leveraged, with SAGEConv (Graph SAGE Convolution) as the encoder. In the SAGEConv reconstruction module, by reconstructing the relationships between transaction graph nodes, the structural features of the nodes were learned, obtaining reconstructed node embedding representations. Ultimately, phishing fraud nodes were further identified by integrating temporal features, basic features, and embedding representations. A real Ethereum dataset was collected for evaluation, and the DA-HGNN model achieved an AUC-ROC (Area Under the Receiver Operating Characteristic Curve) of 0.994, a Recall of 0.995, and an F1-score of 0.994, outperforming existing methods and baseline models.

## 1. Introduction

With the rapid development of blockchain technology, Ethereum, as a decentralized platform supporting smart contracts, has become an essential infrastructure for cryptocurrencies and decentralized applications [1]. Ethereum has driven innovations in financial technology and has had profound economic and societal impacts globally, ranking second only to Bitcoin in the cryptocurrency market share [2]. However, as the Ethereum ecosystem expands, security threats such as phishing scams are becoming increasingly severe.

Phishing is a well-known cybercrime where fraudsters lure victims into revealing sensitive data and impersonate trustworthy entities to steal funds. This tactic has also spread to the blockchain ecosystem [3]. Unlike traditional phishing scammers who prefer creating fake websites to gather users’ personal information, Ethereum scammers tend to entice victims to transfer Ether or grant permissions to phishing accounts, thereby obtaining substantial returns [4]. Research organizations have identified over 5000 phishing accounts in Ethereum, some of which have been active in the past two to three years, with stolen assets amounting to tens of millions of dollars [5,6]. In 2023, a phishing attack targeting the Ethereum Denver conference led to the theft of over USD 300,000 in Ether. Criminals even paid for Google ads to rank their fraudulent sites higher than the official ETHDenver website [7]. Many blockchain industry professionals have fallen prey to these scams, and the general public is even more vulnerable. Thus, developing effective phishing detection technologies to identify and prevent these malicious activities has become an urgent task in blockchain security research.

Previous studies on detecting Ethereum phishing scams primarily relied on analyzing transaction records and account behaviors, using rule-based methods or traditional machine learning techniques to identify suspicious activities [8]. However, these methods often require extensive manual feature engineering and struggle to adapt to complex and evolving attack strategies. In 2020, graph neural networks were first introduced to Ethereum phishing scam detection, demonstrating potential in handling large-scale transaction data and mining complex account relationships [9]. By efficiently utilizing Ethereum’s graph-structured data, these networks can more accurately capture interactions between accounts and potential fraudulent behaviors. Furthermore, employing formal methods to verify the behavior of smart contracts within their execution environments can significantly minimize the risk of faults and errors while also avoiding potential associated costs [10,11]. Despite some progress, detecting Ethereum phishing scams still faces many challenges. Firstly, the data samples of phishing accounts scraped from the Ethereum network are imbalanced, and the network exhibits significant heterogeneity [12]. However, no researchers have yet studied and solved these problems via data augmentation technology combined with graph neural networks. Secondly, most existing methods based on transaction record analysis use graph embedding technologies, which do not fully utilize transaction data and rely too much on the spatial structure of nodes, neglecting the importance of time series.

Therefore, this study proposes an Ethereum phishing scam detection method based on DA-HGNN (Data Augmentation Method and Hybrid Graph Neural Network Model). Initially, an Ethereum transaction network is constructed using the scraped phishing nodes and their transaction information. Then, basic features for nodes are designed, and a sliding window sampling method for data augmentation based on transaction data is employed, thereby obtaining time series features. Via data augmentation, the nodes retain their original topological relationships, which helps address the issue of data imbalance and mitigates the graph’s heterogeneity. Subsequently, using the GAE (Graph Autoencoder) concept, SAGEConv (Graph SAGE Convolution) is adopted as the encoder. In the SAGEConv Reconstruction Module, using the enhanced transaction graph, the structural features of nodes are learned, and the reconstructed node embedding representation is obtained. Simultaneously, models sensitive to time series features are used to discover the temporal relationships between features, resulting in the derivation of temporal features. Ultimately, the temporal features, basic features, and embedding representations are integrated to identify phishing nodes further. The main contributions of this study can be summarized as follows:

(1) By solving the sample imbalance issue via data augmentation and using SAGEConv to reconstruct the enhanced transaction graph and data, the embedding representation of nodes is obtained, reducing the transaction network’s heterogeneity while improving model performance.
(2) Conv1D (One-Dimensional Convolutional neural network) and GRU-MHA (GRU- Multi-Head Attention) are used to discover the intrinsic relationships between data in time series features, obtaining temporal features.
(3) The proposed DA-HGNN model integrates temporal features, basic features, and embedding representations to enhance the performance of Ethereum phishing scam detection. Extensive experiments were conducted on real-world Ethereum phishing scam datasets, and the model’s effectiveness was proven by comparison with existing methods.

The remainder of this paper is organized as follows. Section 2 summarizes recent research on Ethereum phishing scam detection. Section 3 introduces the technical details and overall detection framework of the proposed DA-HGNN model. Section 4 presents the evaluation metrics for assessing phishing detection performance. Section 5 details the experimental setup and evaluates the proposed model’s performance in detecting phishing on Ethereum. Finally, Section 6 concludes the paper and discusses future work.

## 2. Related Work

In this section, recent research on Ethereum phishing scam detection is summarized. According to the different ways of obtaining node features, the research methods are divided into three categories: manual feature extraction, feature extraction based on embedding algorithms, and hybrid feature fusion model.

### 2.1. Manual Feature Extraction Methods

Chen et al. [13] constructed a transaction graph using transaction data from Ethereum and known phishing addresses and proposed a graph-based cascading feature extraction method. To train classification models, they built a second-order transaction graph for the target accounts. They employed ensemble algorithms that combined multiple baseline models for sample and feature training to address class imbalance, selecting LightGBM as the final classifier. This method proved effective in identifying Ethereum phishing accounts. Wen et al. [14] proposed two phishing account detection methods: one that is based on a feature learning framework and the other that focuses on transaction record insertion. In the feature learning framework, they extracted 25 account features, including account balance, transaction count, average transaction amount, and network features like in-degree and out-degree from transaction records. These features were combined with machine learning models for phishing activity detection and validated experimentally for their effectiveness. Additionally, to test the robustness of the detection system, they designed a framework that could obscure phishing activities by inserting malicious transactions into accounts. Experiments demonstrated that both frameworks achieved good results in Ethereum phishing scam detection tasks.

### 2.2. Feature Extraction Methods Based on Embedding Algorithms

Feature extraction methods based on embedding algorithms obtain node embeddings by constructing transaction graphs. Grover et al. [15] developed the Node2Vec method, which learns to map nodes to a low-dimensional feature space while preserving the structural features of the node network neighborhoods, thus effectively exploring different neighbors. Using this approach, Yuan et al. [16] applied Node2Vec to detect network phishing scam accounts. They used node embeddings generated by Node2Vec, which preserved the local structural features of the data, and experiments showed that the detection performance using Node2Vec significantly outperformed other methods. Wu et al. [17] developed a new network embedding algorithm called trans2vec, an improved version of Node2Vec that is better suited for handling transaction graphs. Unlike Node2Vec, trans2vec determines the weight of edges based on the total number of transactions between two nodes and the time of the last transaction rather than relying on random selection. Furthermore, trans2vec uses transaction information to calculate edge weights, making it more suitable for Ethereum phishing detection needs. Experimental results showed that trans2vec performed better in detecting phishing scam accounts than Node2Vec, demonstrating its effectiveness and efficiency in handling transactional data. In 2023, Lin et al. [7] introduced Phish2vec, which combined TSG (Temporal-based Sequences Generator) and HSG (Heterogeneous-based Sequences Generator) to optimize transaction representation learning based on transaction timing constraints and account type diversity. Additionally, a novel SBS (Statistics-Based Sampling) method was proposed to address potential label leakage issues, further refining the learning process. Various classifiers used in the experiments confirmed the effectiveness and stability of Phish2vec relative to other methods.

### 2.3. Hybrid Feature Fusion Model Methods

Li et al. [18] proposed a hybrid model combining LSTM (Long Short-Term Memory) and GCN (Graph Convolutional Networks) to analyze the temporal and structural features of the Ethereum transaction graph. The authors used LSTM to capture the temporal features of edges in the transaction graph and then aggregated these temporal edge features via an attention mechanism to enhance node representations. These node representations were then input into the GCN module to extract their structural features. Ultimately, the model integrated multiple groups of features to improve the identification accuracy of phishing addresses. This method effectively fused temporal sequence and graph structure information, providing an efficient solution for phishing scam detection, with an AUC-ROC (Area Under the Receiver Operating Characteristic Curve) value reaching 92.8%. Wen et al. [19] first used data augmentation in an Ethereum phishing network dataset with sample imbalance. They proposed a hybrid deep learning model, LBPS (LSTM-FCN and BP neural network-based Phishing Scam accounts detection model), which used a BP neural network to extract the implicit relationships between features and combine it with LSTM-FCN to capture the temporal features of transaction records. Their model combined these two groups of features as the final node feature representation. Experimental results showed that LBPS outperformed baseline model methods and existing methods, with an F1 score of 97.86%. However, it did not utilize graph neural networks for further processing.

## 3. Proposed Method

**Figure 1:**
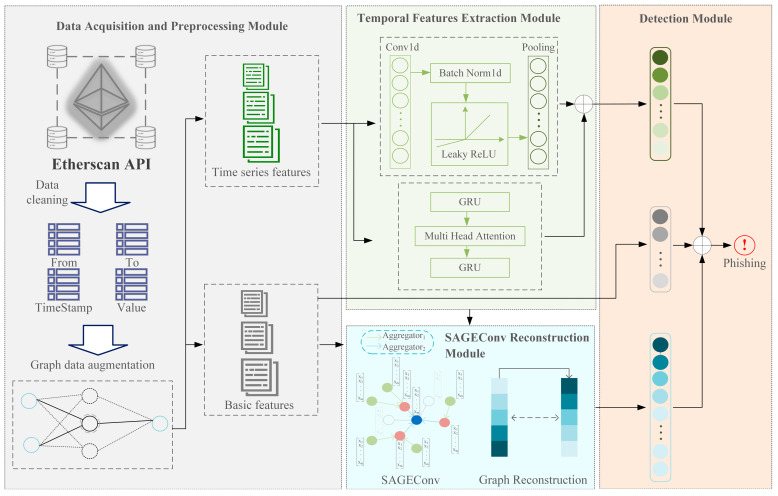
The overall architecture of DA-HGNN.

This section provides a detailed description of the proposed method. Figure 1 illustrates four modules: Data Acquisition and Preprocessing Module, Temporal Features Extraction Module, SAGEConv Reconstruction, and Detection Module.

### 3.1. Data Acquisition and Preprocessing Module

#### 3.1.1. Data Acquisition

Data were obtained from the authoritative Ethereum blockchain explorer, etherscan.io. Due to the public nature of blockchain platforms, every user can access comprehensive transactional, contract, and address data via this explorer. Utilizing the Application Programming Interface (API) provided by etherscan.io, transaction data involving 2761 labeled phishing nodes and their first-order neighbors were acquired. Ultimately, 348,159 transaction records and 121,425 Ethereum addresses were collected.

#### 3.1.2. Data Cleaning

We performed data processing to reduce data redundancy and obtain more representative features, following the data cleaning condition outlined in [18]. First, we kept transaction data from 1 January 2022 to 1 April 2024 to obtain the latest phishing node activity information. Then, addresses with less than 5 or more than 1000 transaction records were deleted, which may be wallets or other normal types of accounts [17,18]. After cleaning, 179,506 transaction records and 66,402 Ethereum addresses were obtained, including 1709 phishing node addresses. These data include four feature columns, From, To, TimeStamp, and Value, representing the transaction sender address, recipient address, transaction timestamp, and transaction amount, respectively.

#### 3.1.3. Data Augmentation

Sampled transaction subgraphs containing 100, 150, and 200 phishing nodes as experimental datasets labeled D1, D2, and D3 from the original transaction graph. Based on the original data, an 11-dimensional feature vector was designed as the basic features for the nodes. These basic features include the total number of transactions, number of transactions sent, number of transactions received, average transaction amount, total transaction amount, average amount sent, average amount received, total amount sent, total amount received, amount of the last transaction sent, and amount of the last transaction received.

Table 1 shows the statistical information for each dataset, indicating the total number of transactions before augmentation. There is a significant disparity in sample numbers between phishing and benign nodes. Hence, a sliding window sampling method addresses the data imbalance issue and then constructs transaction time series data consisting of n timesteps, where n denotes the sliding window size [19]. Each time step represents a time series feature of the node, including transaction amount, transaction direction, and transaction timestamp. For transaction time series exceeding n timesteps, multiple rounds of sampling are conducted, with the window sliding forward by [n/2] steps after each round. Each feature value is set to zero for time series shorter than n timesteps by default. Via experimentation, it was found that a timestep length of 4 tends to balance the data samples; thus, the sliding window was set to 4. Since phishing nodes often initiate numerous transactions, they can generate more time series samples than benign nodes. It is important to note that the augmented samples inherit the topological structure of the original samples. Table 1 also presents the augmented sample numbers, which are roughly balanced, resolving the data imbalance issue.

### 3.2. Temporal Features Extraction Module

This module will process the obtained time series features. Time series features are transaction records arranged chronologically, obtained by data augmentation via sliding window sampling. Capturing the temporal relationships between features and deeply mining effective features play a crucial role in the ultimate classification performance.

Xiao et al. [20] proposed a novel RTFN (Robust Temporal Feature Network) for feature extraction in time series classification, which includes a TFN (Temporal Feature Network) and an LSTM-based Attention Network (LSTMaN). The TFN, a residual structure with multiple convolutional layers, is a local feature extraction network that mines sufficient local features from the data. It utilizes a convolutional block, Conv1D, consisting of a one-dimensional CNN (Convolutional Neural Networks) module, batch normalization, and LeakReLU (leaky rectified linear unit) activation function [21], defined as

(1) $${Out}_{Conv1D}=F_{LeakReLU}\left(F_{BN}\left(F_{conv}\left(C_{x}\right)\right)\right)$$

where ${Out}_{Conv1D}$ and $C_{x}$ represent the output and input of the Conv1D module, respectively. $F_{LeakReLU}$, $F_{BN}$, and $F_{conv}$ denote the LeakReLU activation, batch normalization, and one-dimensional CNN function.

Batch normalization normalizes the input data of each batch to maintain a stable input distribution across layers, helping to prevent gradient vanishing or explosion issues, thus accelerating the convergence of the network. It also enhances its capability in supervised classification and unsupervised clustering [20].

Unlike the ReLU (rectified linear unit), which only considers positive numbers, LeakReLU processes both positive and negative numbers, helping to reduce feature loss during data transmission. LeakReLU is defined as

(2) $$F_{LeakReLU}\left(L_{x}\right)=\left\{\begin{array}{c} L_{x},L_{x}\;\geq 0\\ {\sigma L}_{x},\;L_{x}<0 \end{array}\right.$$

where $L_{x}$ is the input to LeakReLU, and σ is the coefficient for negative inputs, which is set to 0.1 in this paper.

Based on RTFN, this study employs Conv1D and GRU-MHA to extract temporal features, as depicted in Figure 1 in the Temporal Features Extraction Module. GRU [22] can selectively update and forget information, effectively modeling the dependencies of time series data over long periods. This allows GRU to capture deep temporal features between nodes. Compared to LSTM, GRU has a simpler structure, making training easier. The multi-head attention can learn different attention distributions, where each head can focus on different points of interest, thus generating richer representations [23]. The average suppresses this situation compared to a single attention head [24]. The multi-head attention formula is as follows:

(3) $$MultiHead\left(Q,K,V\right)=Concat\left({head}_{1},\ldots ,{head}_{h}\right)W^{O}$$

(4) $${head}_{i}=Attention\left({QW}_{i}^{Q},{KW}_{i}^{K},{VW}_{i}^{V}\right)$$

where Q,K,V are the input matrices for the multi-head attention mechanism, representing queries, keys, and values, respectively; $W^{Q},W^{K},W^{V},W^{O}$ are used to project the queries, keys, values, and the output of the multi-head attention into their respective spaces.

And each ${head}_{i}$ uses scaled dot-product attention, which is computed as

(5) $$Attention\left(Q,K,V\right)=softmax\left(\frac{QK^{T}}{\sqrt{d_{k}}}\right)V$$

where $d_{k}$ represents the dimension of the keys. It is used to scale the dot product in the attention mechanism to help stabilize the gradients during training. The softmax is applied to the weights (scaled dot products) to compute attention scores, determining the focus on each value vector V. Input the time series features into Conv1D and GRU-MHA, respectively, and then splice the output results of these two models together as the output of the Temporal Features Extraction Module, that is, temporal features.

### 3.3. SAGEConv Reconstruction Module

This module aims to extract topological structure information between nodes by reconstructing the transaction graph using SAGEConv. The output of the Temporal Features Extraction Module and basic features are spliced together as a node embedding representation. Using the GAE concept [25], it is input into a model with SAGEConv as the encoder to learn the topological structure information of nodes, thereby obtaining the final embedding representation of the nodes. Sun et al. [26] also used the concept of GAE. The difference is that we used SAGEConv as the encoder, which is more suitable for transaction networks with strong heterogeneity such as Ethereum.

SAGEConv is a graph neural network layer designed to generate node embeddings by sampling and aggregating features from the local neighborhood of nodes [27]. Phishing nodes often engage in numerous transactions, by sampling and aggregating neighbor information of phishing nodes using SAGEConv, node embeddings that are distinct and more representative compared to benign nodes are obtained, which benefits further classification tasks. The aggregation process is illustrated in Figure 1. The basic formula for SAGEConv is

(6) $${\;\;\;\;h}_{i}^{\left(l+1\right)}=\rho \left(W\cdot AGGREGATE\left(\left\{h_{j}^{\left(l\right)},\forall j\in N\left(i\right)\right\}\right)+B\cdot h_{i}^{\left(l\right)}\right)$$

where $h_{i}^{\left(l\right)}$ is the feature vector of node i at layer l, $N\left(i\right)$ represents the set of neighbors of node i; AGGREGATE is a function that aggregates features from the neighborhood; W and B are trainable weight matrices; ρ is a non-linear activation function.

### 3.4. Detection Module

This module aims to classify nodes to distinguish between phishing and benign nodes. Three sets of features are spliced together to obtain a complete representation of the node: the basic features obtained from the Data Acquisition and Preprocessing Module, the temporal features extracted from the Temporal Features Extraction Module, and the embedded representation generated by the SAGEConv Reconstruction Module. This complete representation is input into a fully connected layer to produce the detection results.

## 4. Metrics

The detection of phishing scams is treated as a binary classification task. Several metrics will be utilized to assess the model’s effectiveness, including Accuracy, Precision, Recall, F1-Score, FPR (False Positive Rate), FNR (False Negative Rate), AUC-ROC, and AUC-PR (Area Under the Precision–Recall Curve). The classifier’s performance improves as the ROC curve approaches the top left corner of the plot. AUC-ROC is a critical metric for evaluating the quality of classifiers, where a higher AUC-ROC value indicates better model performance. The PR (Precision–Recall) curve depicts the trade-off between Precision and Recall for the model. AUC-PR measures the model’s ability to capture true positives while maintaining high precision. These metrics depend on four terms: TP (True Positive), TN (True Negative), FN (False Negative), and FP (False Positive) [28]. The specific formulas are as follows:

(7) $$Precision=\frac{TP}{TP+FP}$$

(8) $$F1-Score=2*\frac{Recall*Precision}{Recall+Precision}$$

(9) $$Recall=\frac{TP}{FN+TP}$$

(10) $$Accuracy=\frac{TP+TN}{TN+FN+TP+FP}$$

(11) $$FPR=\frac{FP}{FP+TN}$$

(12) $$FNR=\frac{FN}{FN+TP}$$

## 5. Experimental Results

In this section, the effectiveness of the proposed model in detecting Ethereum phishing scams will be evaluated. The experiments were conducted using the Python programming language. The runtime and testing environment included a 12th Gen Intel (R) Core (TM) i7-12700F CPU at 2.10 GHz, NVIDIA GeForce RTX 4090 GPU, 32.0 GB RAM, Windows 11 Professional operating system, and PyTorch version 1.13.1. Information about the dataset can be found in Section 3. In the experiments, 70% of the dataset was used as the training set and the remainder as the test set. The DA-HGNN model was trained with 100 epochs, a learning rate of 0.001, and a batch size of 256, using the Adam optimizer and Cross Entropy Loss as the loss function.

### 5.1. Comparison Methods

To demonstrate the effectiveness of the proposed DA-HGNN model in detecting Ethereum phishing scams, the performance of the HGNN model before data augmentation was first compared with that of the DA-HGNN model after augmentation. Subsequently, extensive comparative analyses were conducted. Comparisons were made between the model and methods within four categories: Graph Neural Networks, Deep Learning, Random Walk, and Machine Learning, all using data after augmentation. The specific methods are as follows:

- SAGEConv [18] is a graph neural network layer that learns node representations on graph data by aggregating neighbor features to update node representations.
- GATv2 (Graph Attention Network Version 2) [29] is an improved version of the GAT (Graph Attention Network) model that learns node representations on graph data via a self-attention mechanism to capture relationships between nodes better.
- GAT [6] is a model that uses an attention mechanism to learn node representations on graph data.
- GCN [30] is a convolutional neural network for graph data to learn node representations.
- CNN [31] is primarily used for processing and analyzing data with a grid structure.
- GRU [32] is a variant of RNN (Recurrent Neural Network) with a simple structure, often used for processing sequence data.
- LSTM [19] is specifically designed to address problems of gradient vanishing and explosion in long sequence data, suitable for processing sequences with long-term dependencies.
- A-CNN (Attention-CNN) [4] combines CNN and attention mechanisms to enhance CNN performance in processing sequence data, especially in tasks like text classification and sequence tagging.
- Node2Vec [16] is a graph embedding technique that maps nodes in a graph to low-dimensional vector spaces for subsequent machine learning tasks.
- Deep Walk [33] is an unsupervised method for learning node representations in graph data, generating node sequences via random walks and using these sequences to learn low-dimensional embeddings.
- LightGBM [34] is an efficient gradient-boosting framework that uses a histogram-based decision tree learning algorithm with fast training speed and high accuracy.
- RF (Random Forest) [35] performs classification or regression tasks by building multiple decision trees and integrating them.
- SVM (Support Vector Machine) [36] performs classification by finding the optimal separation boundary between different classes in a dataset.

### 5.2. Training Process Analysis

**Figure 2:**
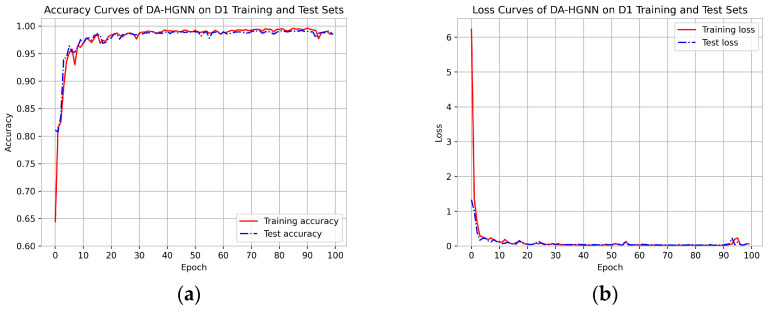
Accuracy and loss curves of DA-HGNN on the D1 dataset. (**a**) Accuracy curves; (**b**) loss curves.

**Figure 3:**
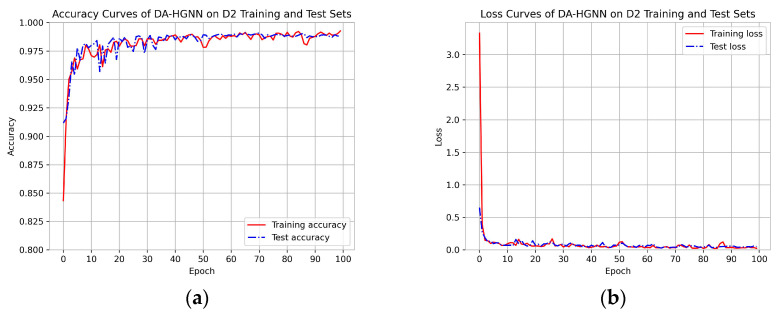
Accuracy and loss curves of DA-HGNN on the D2 dataset. (**a**) Accuracy curves; (**b**) loss curves.

**Figure 4:**
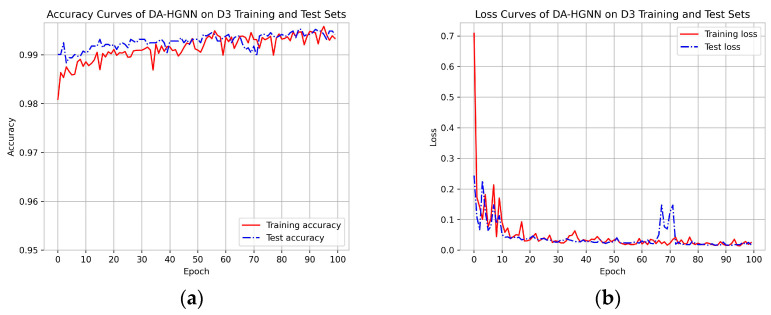
Accuracy and loss curves of DA-HGNN on the D3 dataset. (**a**) Accuracy curves; (**b**) loss curves.

Figure 2, Figure 3 and Figure 4a,b show the accuracy and loss curves of DA-HGNN on the D1, D2, and D3 datasets, respectively. As shown in the figures, after 100 training epochs, the accuracy and loss curves on the test set have stabilized, indicating that the model has converged and reached its optimal state. By observing Figure 2, Figure 3 and Figure 4b, it can be seen that as the dataset size increases, the loss decreases at a faster rate. This indicates that the model can achieve optimal performance with fewer training epochs on larger datasets. 

**Figure 5:**
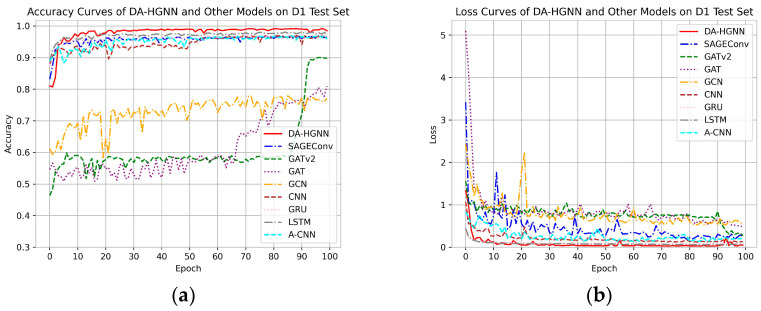
Accuracy and loss curves of DA-HGNN and different models on the D1 test set. (**a**) Accuracy curves; (**b**) loss curves.

**Figure 6:**
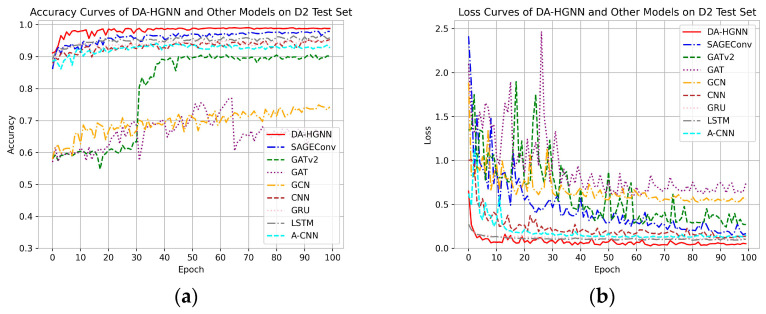
Accuracy and loss curves of DA-HGNN and different models on the D2 test set. (**a**) Accuracy curves; (**b**) loss curves.

**Figure 7:**
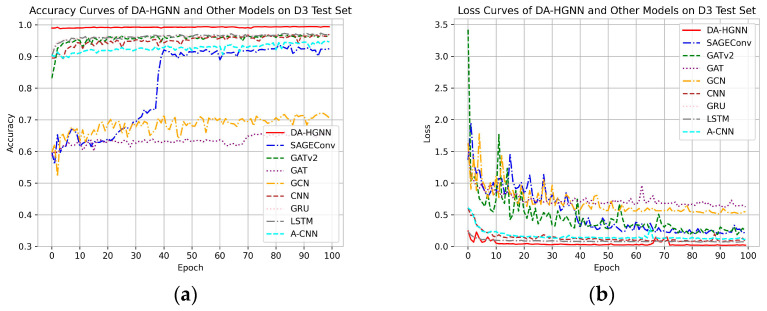
Accuracy and loss curves of DA-HGNN and different models on the D3 test set. (**a**) Accuracy curves; (**b**) loss curves.

Figure 5, Figure 6 and Figure 7a,b display the accuracy and loss comparison curves of DA-HGNN with other models. From these figures, the following conclusions can be drawn:

In Figure 5, Figure 6 and Figure 7a,b, it is evident that after convergence, the accuracy curve of DA-HGNN is higher than that of other models, while its loss curve is the lowest. This indicates that DA-HGNN outperforms other models in terms of performance.

From Figure 7, it can be observed that when the model is applied to the D3 dataset, its convergence speed is faster than that of other models, suggesting that DA-HGNN is better at capturing the features within the data.

Additionally, by further examining Figure 5 and Figure 6, it can be found that the convergence speed increases as the dataset size grows. This demonstrates that DA-HGNN possesses high stability and robustness, maintaining excellent performance in complex data environments. 

### 5.3. Effectiveness Evaluation

This section evaluates the performance of all comparison methods in detecting Ethereum phishing scams on the network. The corresponding results are displayed in Table 2. The following conclusions can be drawn:

(1) Table 2 shows that although HGNN achieves a Precision of 100.0% on dataset D1, surpassing both DA-HGNN and the control group, its other metrics are relatively lower. This indicates that sample imbalance significantly impacts model performance, possibly leading the model to overly focus on certain categories during training, thus reducing its generalization ability on the test set.
(2) DA-HGNN generally performs best across all datasets. Notably, dataset D3 achieves a Precision of 99.3%, an F1-Score of 99.4%, a Recall of 99.5%, and an AUC-ROC score of 99.4%, demonstrating optimal performance and its capability to handle complex transaction networks. The method closest to DA-HGNN in performance is SAGEConv, which achieves an AUC-ROC of 98.2% on dataset D3. Metrics for Deep Learning methods are above 90%, but compared to these, the performance of other Graph Neural Networks methods, except for SAGEConv, is poorer. This indicates that Deep Learning methods have advantages in handling balanced datasets but do not reach optimal performance due to a lack of learning of the topological relationships between nodes.
(3) Similar high-performance methods like LSTM show good results on the smaller dataset D1, while SAGEConv performs better on the larger dataset D3. However, DA-HGNN achieves better performance across all three datasets. After calculations, the maximum fluctuation of the model between the same metrics across different datasets does not exceed 0.5%, and the fluctuation between different metrics within the same dataset does not exceed 0.6%. This indicates the strong robustness of the model, which can stably adapt to different datasets and metric evaluation systems.
(4) Within the Random Walk methods, Node2Vec performs the best. However, on dataset D1, its F1-Score and AUC-ROC are still 18.2% and 8.8% lower than the DA-HGNN model. Node2Vec performs slightly better than Deep Walk on most metrics, likely due to the optimized random walk strategy of Node2Vec that more effectively explores the neighborhood structure of the network. In contrast, Deep Walk relies merely on simple random walks.
(5) LightGBM and RF outperform SVM in Precision, F1-Score, Recall, and AUC-ROC, possibly because they handle large-scale datasets and complex feature spaces better. Among the machine learning methods, LightGBM shows superior performance with the highest Recall of 98.5% in the control group, indicating its effectiveness in identifying positive classes. Although machine learning methods excel on certain performance metrics, they generally do not match deep learning methods’ stability and generalization capabilities.

**Figure 8:**
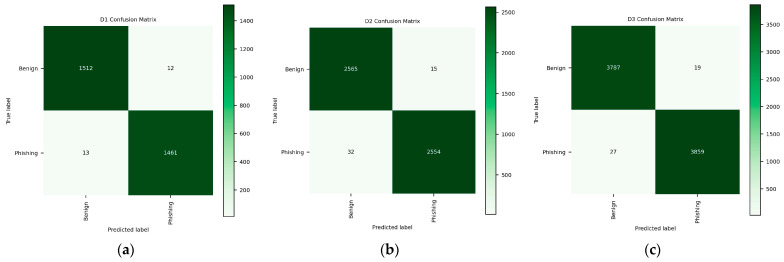
Confusion matrix of DA-HGNN on three datasets. (**a**) Confusion matrix on the D1 test set; (**b**) confusion matrix on the D2 test set; (**c**) confusion matrix on the D3 test set.

This study uses a confusion matrix to further evaluate the performance of DA-HGNN. Figure 8 shows the confusion matrix results of DA-HGNN on the D1, D2, and D3 test sets. Each column of the confusion matrix represents the predicted category, and the total number of each column represents the amount of data predicted for that category; each row represents the true category of the data, and the total amount of data in each row represents the number of data instances of that category. The numbers on the diagonal represent the number of correctly classified samples, while the numbers outside the diagonal represent the number of samples where the predicted classification is inconsistent with the true classification, that is, the number of incorrect classifications. By observing the three confusion matrices, we can find that the number of samples on the diagonal is much more than the number outside the diagonal, which shows that DA-HGNN performs well.

Furthermore, based on the confusion matrix presented in Figure 8, the FPR and FNR values for three datasets were calculated. The FPR and FNR values for dataset D1 were 0.0079 and 0.0088, respectively; for D2 were 0.0058 and 0.0123, and for D3 were 0.005 and 0.007, respectively. The experimental results indicate that the DA-HGNN model achieves exceptionally low FPR and FNR across all three datasets, with the lowest values observed in the D3 dataset. A lower FPR signifies fewer normal transactions being incorrectly flagged as fraudulent, which helps reduce the time and resources spent on handling false alarms. A lower FNR indicates effective identification and marking of genuine phishing attempts, ensuring that nearly all malicious activities are detected and addressed.

**Figure 9:**
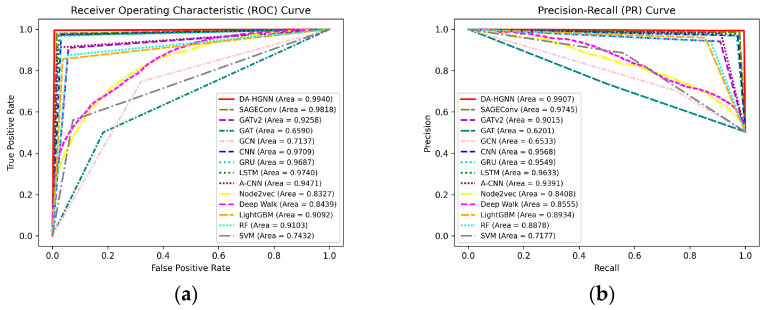
ROC and PR curves of DA-HGNN and different models on the D3 dataset. (**a**) ROC curves; (**b**) PR curves.

Figure 9a,b show the ROC and PR curves of different models on dataset D3. It can be observed that DA-HGNN achieves an ROC curve AUC of 0.9940 and a PR curve AUC of 0.9907, demonstrating its ability to differentiate between positive and negative classes more accurately. Section 3 mentions that DA-HGNN incorporates CNN, GRU, and SAGEConv as key model components. It is evident from the figures that when CNN, GRU, and SAGEConv appear alone, the effects are slightly inferior. This study uses CNN and GRU to capture the relationships between transaction amounts and timings, while SAGEConv captures the topological relationships between nodes. Ultimately, these are combined with the basic features of the nodes to complete the detection, thus allowing DA-HGNN to achieve optimal performance. Compared to Graph Neural Networks, Deep Learning, Random Walk, and Machine Learning, DA-HGNN more effectively integrates Precision and Recall in handling classification tasks, particularly evidenced by its high AUC value on the PR curve, highlighting its advantage in predicting positive classes.

### 5.4. Ablation Study 

**Figure 10:**
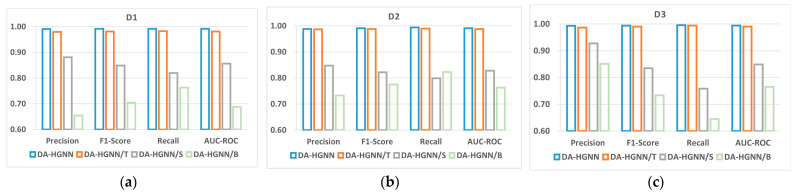
Ablation experimental results of different features in three datasets. (**a**) Ablation experiment on the D1; (**b**) ablation experiment on the D2; (**c**) ablation experiment on the D3.

To evaluate the contribution of features from each module, the Temporal Features Extraction Module (DA-HGNN/T) was individually removed, the SAGEConv Reconstruction Module (DA-HGNN/S), and the basic features (DA-HGNN/B). The impact of removing these modules on DA-HGNN is shown in Figure 10, from which the following conclusions can be drawn:

(1) It is evident from the figure that the performance of DA-HGNN/B significantly declines after the removal of basic features, with its AUC-ROC values decreasing by 30.5%, 22.8%, and 22.9% in datasets D1, D2, and D3, respectively. This indicates that the basic features designed play a crucial role in the detection task. Particularly in blockchain platforms like Ethereum, where user anonymity prevails, nodes inherently lack features, making basic features even more essential. However, Refs. [13,14] have shown that relying solely on manually designed features is insufficient to enhance detection performance further. Integrating structural relationships and temporal information between nodes into the feature design process is the main challenge. DA-HGNN compensates for these deficiencies, thereby achieving optimal performance.
(2) DA-HGNN/T has little impact on the performance of the model. Its Precision, F1-score, Recall, and AUC-ROC on the D1 dataset are 98.0%, 98.1%, 98.2% and 98.1%, respectively. Compared with DA-HGNN, the maximum improvement is only 1.1%. However, it is noteworthy that the overall performance metrics of DA-HGNN/T have already reached over 98.0%, making further improvements challenging. In a highly optimized model, the Temporal Features Extraction Module fine-tunes the model by capturing the underlying temporal patterns of transaction amounts. Thus, even less than 1.1% improvement is significant for the model.
(3) After removing the SAGEConv Reconstruction Module, the performance of DA-HGNN/S on dataset D3 decreases by 14.5% in terms of AUC-ROC. This module mines the topological structure information between nodes to obtain a more comprehensive node embedding representation. This embedding representation, in conjunction with basic and temporal features, is crucial for enhancing the overall performance of the DA-HGNN model.

### 5.5. Sensitivity Analysis

This section explores the impact of the number of multi-head attention heads and the size of the graph embedding dimension on the performance of DA-HGNN.

**Figure 11:**
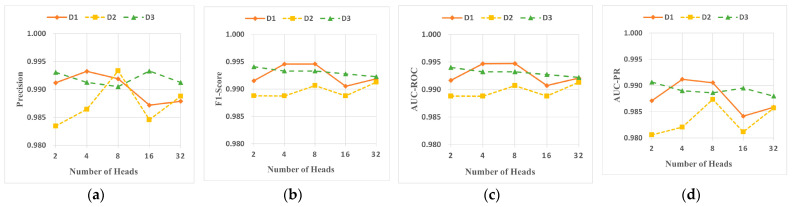
Sensitivity analysis of multi-head attention heads on DA-HGNN. (**a**) Precision; (**b**) F1-Score; (**c**) AUC-ROC; (**d**) AUC-PR.

**Figure 12:**
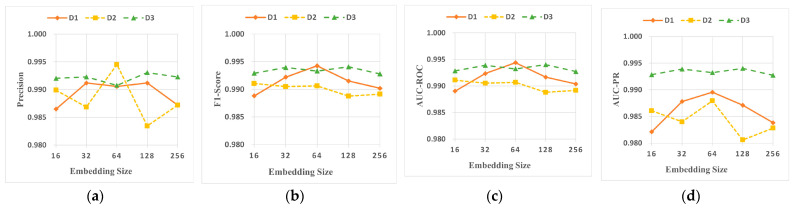
Sensitivity analysis of embedding dimension size on DA-HGNN. (**a**) Precision; (**b**) F1-Score; (**c**) AUC-ROC; (**d**) AUC-PR.

Figure 11 illustrates the changes in Precision, F1-Score, AUC-ROC, and AUC-PR scores of DA-HGNN across three datasets as the number of attention heads increases from 2 to 32. In contrast, Figure 12 shows how these performance metrics change as the embedding dimensions increase from 16 to 256. From the observations of Figure 11 and Figure 12, the following conclusions can be drawn:

(1) Figure 11 shows that the variation in metrics for the D3 dataset is less significant than for the D1 and D2 datasets. This indicates that DA-HGNN requires fewer attention heads to perform well when dealing with large datasets. A similar conclusion can be drawn from Figure 12, where the fluctuation in metrics for the D3 dataset is at most 0.3% when the embedding dimensions change, demonstrating that DA-HGNN possesses strong robustness to changes in the number of attention heads and embedding dimensions when processing large datasets.
(2) Observing Figure 11, it is found that increasing the number of attention heads does not always lead to better performance. For the D2 dataset, performance trends upward as the number of attention heads increases from 2 to 8. However, performance declines when it increases to 16. Therefore, selecting the appropriate number of attention heads is crucial for enhancing model performance. A similar trend can be observed in Figure 12. The D1 dataset shows a more distinct trend, where metrics generally increase steadily as the embedding dimensions rise from 16 to 64, except for precision, which shows an initial increase followed by a slight decrease. With the continuous increase in embedding dimensions, performance notably decreases. Excessively high vector dimensions may lead to overfitting and significantly increase computational costs; conversely, too low dimensions may reduce representational performance. Thus, selecting the proper embedding dimensions is also crucial.
(3) Combining the above analysis, it is concluded that DA-HGNN demonstrates good fault tolerance in performance when handling larger datasets, whether adjusting the number of heads in the attention mechanism or the dimensions of the embedding vectors. This robustness to parameter changes implies that it can effectively resist performance fluctuations in the presence of different data noise, reducing the complexity and uncertainty in the model-tuning process.

### 5.6. Scalability Analysis

**Figure 13:**
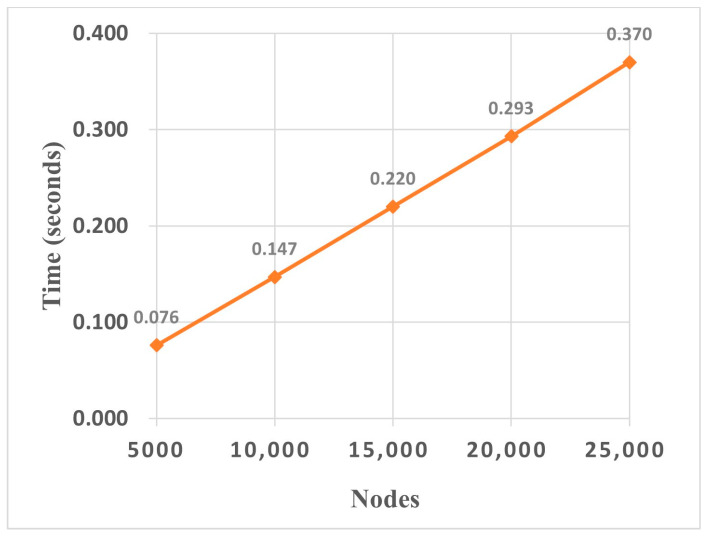
Running time of DA-HGNN under different numbers of nodes.

This module conducted an efficiency analysis of the DA-HGNN to evaluate its scalability. The experiments utilized a trained DA-HGNN model with a size of 554 KB. The number of test nodes was increased from 5000 to 25,000. For each node scale, 500 independent experiments were conducted to calculate the average runtime. The runtime results are shown in Figure 13. It can be observed that the runtime of DA-HGNN has a linear relationship with the number of nodes, which is acceptable in practice. Moreover, it only takes 0.370 s to process 25,000 nodes, indicating a rapid response time. Therefore, it can be concluded that DA-HGNN is a scalable approach, suitable for application in large-scale real-time systems.

## 6. Conclusions

This paper proposes an Ethereum phishing scam detection method based on data augmentation and a hybrid graph neural network. Initially, the method addresses data imbalance by introducing a sliding window sampling technique. Subsequently, Conv1D and GRU-MHA capture the intrinsic relationships between transaction features, mining sufficient local features and obtaining temporal features. Next, learn deep structural features by using SAGEConv as an encoder and combining node topological structure information, basic features, and temporal features to achieve a reconstructed node embedding representation. Finally, by integrating basic features, temporal features, and embedding representations, the effectiveness of Ethereum phishing scam detection is further enhanced. Experimental results demonstrate that the performance of DA-HGNN surpasses existing methods.

Due to the extensive scale of the transaction graph, this study was limited to experiments conducted on extracted subgraphs. In the future, it is hoped that experiments will be performed on more authoritative and larger-scale data to evaluate the scalability and robustness of the model. Additionally, it is anticipated that this study will attract the attention of more researchers and practitioners in blockchain platforms, thereby promoting research innovation in the field of blockchain data security and fraud detection.

## Figures and Tables

**Table 1:** Statistics of experimental datasets.

Dataset	Phishing	Benign	Total Transactions	Augment Phishing	Augment Benign
D1	100	3798	9265	5004	4989
D2	150	6433	16,441	8620	8597
D3	200	9533	23,448	12,843	12,794

**Table 2:** Performance comparison results on three datasets in w.r.t. Precision, F1-score, Recall, and AUC-ROC.

Method	Dataset	D1				D2				D3			
	Metric	Precision	F1-Score	Recall	AUC-ROC	Precision	F1-Score	Recall	AUC-ROC	Precision	F1-Score	Recall	AUC-ROC
Graph Neural Networks	SAGEConv	0.975	0.965	0.955	0.966	0.985	0.980	0.975	0.980	0.984	0.982	0.979	0.982
	GATv2	0.953	0.889	0.832	0.896	0.917	0.899	0.880	0.901	0.941	0.925	0.909	0.926
	GAT	0.775	0.818	0.866	0.811	0.681	0.734	0.796	0.714	0.736	0.596	0.501	0.659
	GCN	0.809	0.762	0.720	0.778	0.735	0.747	0.760	0.744	0.704	0.725	0.747	0.714
Deep Learning	CNN	0.973	0.962	0.950	0.962	0.939	0.943	0.947	0.943	0.969	0.971	0.973	0.971
	GRU	0.968	0.971	0.974	0.971	0.952	0.952	0.953	0.953	0.970	0.969	0.968	0.969
	LSTM	0.982	0.981	0.980	0.981	0.953	0.962	0.972	0.962	0.977	0.974	0.972	0.974
	A-CNN	0.962	0.962	0.962	0.963	0.953	0.926	0.902	0.929	0.981	0.945	0.912	0.947
Random Walk	Node2Vec	0.811	0.810	0.809	0.904	0.764	0.756	0.748	0.838	0.755	0.752	0.750	0.833
	Deep Walk	0.791	0.797	0.802	0.896	0.741	0.753	0.766	0.832	0.763	0.734	0.708	0.844
Machine Learning	LightGBM	0.933	0.906	0.882	0.910	0.893	0.937	0.985	0.934	0.959	0.904	0.855	0.909
	RF	0.932	0.916	0.900	0.918	0.941	0.904	0.870	0.908	0.943	0.907	0.875	0.910
	SVM	0.909	0.790	0.699	0.816	0.852	0.705	0.602	0.749	0.885	0.686	0.560	0.743
Ours	HGNN	**1.000**	0.723	0.567	0.783	0.974	0.881	0.804	0.902	0.894	0.857	0.824	0.911
	DA-HGNN	0.991	**0.992**	**0.992**	**0.992**	**0.988**	**0.991**	**0.994**	**0.991**	**0.993**	**0.994**	**0.995**	**0.994**

## Data Availability

The dataset used in this study can be accessed via the following link: https://github.com/MDBChain/Phishing-Detection-Dataset (accessed on 14 June 2024).
